# Near Infrared Imaging and Photothermal Ablation of Vascular Inflammation Using Single-Walled Carbon Nanotubes

**DOI:** 10.1161/JAHA.112.002568

**Published:** 2012-12-19

**Authors:** Hisanori Kosuge, Sarah P. Sherlock, Toshiro Kitagawa, Rajesh Dash, Joshua T. Robinson, Hongjie Dai, Michael V. McConnell

**Affiliations:** Division of Cardiovascular Medicine, Stanford University, Stanford, CA (H.K., T.K., R.D., M.V.M.); Department of Chemistry, Stanford University, Stanford, CA (S.P.S., J.T.R., H.D.)

**Keywords:** atherosclerosis, imaging, inflammation, macrophage, photothermal therapy

## Abstract

**Background:**

Macrophages are critical contributors to atherosclerosis. Single-walled carbon nanotubes (SWNTs) show promising properties for cellular imaging and thermal therapy, which may have application to vascular macrophages.

**Methods and Results:**

In vitro uptake and photothermal destruction of mouse macrophage cells (RAW264.7) were performed with SWNTs (14.7 nmol/L) exposed to an 808-nm light source. SWNTs were taken up by 94±6% of macrophages, and light exposure induced 93±3% cell death. In vivo vascular macrophage uptake and ablation were then investigated in carotid-ligated FVB mice (n=33) after induction of hyperlipidemia and diabetes. Two weeks postligation, near-infrared fluorescence (NIRF) carotid imaging (n=12) was performed with SWNT-Cy5.5 (8 nmol of Cy5.5) given via the tail vein. Photothermal heating and macrophage apoptosis were evaluated on freshly excised carotid arteries (n=21). NIRF of SWNTs showed higher signal intensity in ligated carotids compared with sham, confirmed by both in situ and ex vivo NIRF imaging (*P*<0.05, ligation versus sham). Immunofluorescence staining showed colocalization of SWNT-Cy5.5 and macrophages in atherosclerotic lesions. Light (808 nm) exposure of freshly excised carotids showed heating and induction of macrophage apoptosis in ligated left carotid arteries with SWNTs, but not in control groups without SWNTs or without light exposure.

**Conclusions:**

Carbon nanotubes accumulate in atherosclerotic macrophages in vivo and provide a multifunctional platform for imaging and photothermal therapy of vascular inflammation.

## Introduction

Cardiovascular disease continues to be the leading cause of death worldwide.^1^ One of the main contributors is the development and disruption of atherosclerotic plaques, which cause ischemia and infarction of downstream tissues. Macrophages are thought to play a major role in plaque formation and rupture,^2–4^ so they are promising targets for detection and therapy of atherosclerosis.

Single-walled carbon nanotubes (SWNTs) have unique physical and chemical properties and have shown promising applications in biological imaging, drug delivery, and thermal ablation.^5–9^ We and others have previously shown that SWNTs exposed to laser light excitation in the biologically transparent near-infrared (NIR) range of 700 to 1100 nm can induce thermal destruction of cancer cells both in vitro and in vivo.^6,8,10^ These SWNTs appear to have advantages over other nanomaterials, as they can achieve thermal destruction of tumors at 10-fold-lower doses and 3-fold-lower power.^10^ Macrophage ablation has potential benefit in atherosclerosis, as prior studies using phototoxic therapy with motexafin lutetium and other agents have shown significant destruction of macrophages in animal models, as well as safety in human studies.^11–15^ However, there are limited data using thermal ablation of macrophages in atherosclerosis.

The aim of the current study was to investigate SWNTs for macrophage imaging and photothermal therapy in a murine model of vascular inflammation.

## Materials and Methods

### Preparation of Single-Walled Carbon Nanotubes

Aqueous SWNT suspensions with and without Cy5.5 fluorescent dye functionalization were prepared by sonicating 0.25 mg/mL of raw HiPCO SWNTs (Carbon Nanotechnologies, Inc, Houston, TX) with 1 mg/mL phospholipid–polyethylene glycol (PL-PEG), as previously reported.^10,16,17^ Cy5.5 concentration and SWNT concentration were calculated using UV-visible absorption spectroscopy at 675 and 808 nm, respectively.^6^

### In Vitro Uptake

Mouse macrophage cells (RAW264.7) were cultured using Chamber slides (Lab-Tek Chamber slides system, Thermo Fisher Scientific Inc, Rochester, NY) in Dulbecco's Modified Eagle's Medium (DMEM; Invitrogen, Carlsbad, CA) containing 50 μg/mL streptomycin, 50 IU/mL penicillin, and 10% fetal bovine serum (FBS) at 37°C and 5% CO_2_. Then cells were incubated with Cy5.5-labeled SWNTs for 24 hours at a concentration of 14.7 nmol/L of SWNTs (2.5 μmol/L of Cy5.5). After washing in PBS, cells were stained with DAPI (Sigma-Aldrich, St Louis, MO) to visualize cell nuclei. Then these cells were imaged by confocal microscopy (Zeiss LSM 510; Carl Zeiss AG, Oberkochen, Germany). The percentage of uptake was measured from 3 samples, with the Cy5.5-positive cells expressed as a percentage of the total cells stained with DAPI. To examine dose-dependent uptake of SWNTs, cells were incubated with Cy5.5-labeled SWNTs for 24 hours at various concentrations of Cy5.5 (0 to 1.2 μmol/L) in 10-cm tissue culture dishes. After incubation, the cells were analyzed by fluorescence-activated cell sorting Calibur (BD Bioscience, San Jose, CA) and FlowJo software (Tree Star Inc, Ashland, OR).

### In Vitro Photothermal Ablation

RAW264.7 cells were incubated with and without 100 nmol/L of SWNTs for 24 hours. Ten million cells (n=4 for each group) were exposed to a continuous 808-nm diode laser light source (RPMC Lasers, Inc, O'Fallon, MO) for 2 minutes at 5 W/cm^2^.^6^ Then cells were immediately stained with Trypan blue and counted by hemocytometer. As additional controls, cells incubated with SWNTs but without laser light exposure were also prepared (n=4). Cell viability was also assessed after 24 hours by MTT assay: exposed cells (1×10^5^/well) were cultured in DMEM containing 50 μg/mL streptomycin, 50 IU/mL penicillin, and 10% FBS at 37°C and 5% CO_2_ for 24 hours; then viability was measured in triplicate of 4 independent experiments with a CellTiter 96 AQ_ueous_ One Solution Cell Proliferation Assay (Promega Corporation, Madison, WI) according to the manufacturer's instructions. Viability was expressed as the optical density of the cells.

### Carotid-Ligation Model

Macrophage-rich atherosclerotic lesions were created in left carotid arteries of FVB mice as described previously.^18–20^ In brief, 8-week-old male mice were fed a high-fat diet containing 40% kcal fat, 1.25% (by weight) cholesterol, and 0.5% (by weight) sodium cholate (D12109; Research Diets, Inc, New Brunswick, NJ). After 1 month on the diet, diabetes was induced by 5 daily intraperitoneal injections of streptozotocin (STZ; 40 mg/kg, Sigma-Aldrich). Two weeks after the initiation of STZ injection, the left common carotid artery was ligated below the bifurcation with the use of 5-0 silk ligature (Ethicon) under 2% inhaled isoflurane (ligation group). Sham operation was performed by passing the suture under the left carotid artery without constricting the artery (sham group). The wound was closed by suture, and the animals were recovered on a warming blanket. The study protocol was approved by the Administrative Panel on Laboratory Animal Care.

### In Vivo Fluorescence Imaging

Two weeks after the operations, 12 mice (8 ligation, 4 sham) were imaged by fluorescence molecular tomography (FMT; FMT 2500 imaging system, Visen, Bedford, MA) at 680/700 nm excitation/emission wavelength. This was done prior to and then 24 and 48 hours after injection of Cy5.5-conjugated SWNTs (8 nmol of Cy5.5/mouse, 0.6 nmol of SWNT/mouse) via the tail vein.

After in vivo fluorescence imaging, the left and right carotid arteries were surgically exposed, and in situ fluorescence imaging was performed on the Maestro imaging system (Cri, Woburn, MA) at 675/690 nm excitation/emission. Then the carotid arteries and aortic arch were carefully removed en bloc followed by ex vivo fluorescence imaging using Maestro. Major organs were also removed and imaged by Maestro to examine the biodistribution of SWNTs.

Images were analyzed by placing regions of interest (ROIs) over the carotid arteries (and ex vivo organs) and calculating average signal intensity divided by exposure time.^21^

### Ex Vivo Intrinsic Near-Infrared Imaging and Photothermal Ablation

Two weeks after carotid ligation, 21 mice were injected with or without 0.6 nmol of SWNTs via tail vein (SWNT [+]: n=11, SWNT [−]: n=10). After 48 hours, 11 left carotid arteries were excised and imaged ex vivo (SWNT [+]: n=6, SWNT [−]: n=5). The intrinsic NIR photoluminescence of SWNTs was captured using a 2D liquid nitrogen cooled InGaAs detector (Princeton Instruments, Trenton, NJ). The excitation source was an 808-nm laser, and light emission was collected from 1100 to 1700 nm. As above, ROIs were placed on the carotid arteries, and total signal intensity divided by exposure time was calculated. Then these 11 intact left carotid arteries were exposed to a continuous external 808-nm laser light source for 5 minutes at 5 W/cm^2^. Temperature of the carotids was monitored during laser ablation using a MikroShot camera (LumaSense Technologies, Santa Clara, CA). The remaining 10 left carotid arteries (SWNT [+]: n=5, SWNT [−]: n=5) were prepared similarly but were not exposed to laser light. All 21 left carotid arteries were then incubated at 37°C for 2 hours in DMEM (containing 50 μg/mL streptomycin, 50 IU/mL penicillin, and 10% FBS and 5% CO_2_) and stained for caspase-3 (see below).

### Histology

#### Immunohistochemical staining

Carotid arteries were cut into two 3-mm sections. These sections were embedded immediately in Optimal Cutting Temperature compound (Sakura Finetek USA, Inc, Torrance, CA) and flash-frozen in liquid nitrogen. Frozen sections (5 μm thick) were fixed in acetone for 10 minutes at 4°C. After washing in PBS, the sections were incubated with monoclonal rat anti-mouse Mac-3 antibody (BD Biosciences, San Jose, CA) or polyclonal rabbit anti-mouse cleaved caspase-3 antibody (Cell Signaling Technology, Inc, Danves, MA) overnight at 4°C. The primary antibodies were detected with biotinylated goat anti-rat IgG or biotinylated goat anti-rabbit IgG. Antigen–antibody conjugates were detected with avidin-biotin-horseradish peroxidase complex (Vector Laboratories, Burlingame, CA) according to the manufacturer's instructions. We used 3-amino-9-ethylcarbazole as chromogen and counterstained sections with hematoxylin. Mac-3 images were analyzed using Image J software (NIH Image, Version 1.43) to quantify the area percentage of neointimal macrophage staining.

#### Immunofluorescence double staining

Immunofluorescence was performed to confirm colocalization of macrophages with SWNT-Cy5.5 after ex vivo fluorescence imaging: carotid sections were incubated with monoclonal rat anti-mouse Mac3 antibody overnight at 4°C, then stained with Alexa Fluor 488–conjugated anti-rat IgG (Molecular Probes, Eugene, OR) at room temperature for 2 hours. Immunofluorescence was also performed to assess colocalization of macrophages or smooth muscle cells with caspase-3-positive cells after ex vivo photothermal ablation. For macrophages, carotid sections were incubated with monoclonal rat anti-mouse Mac3 antibody and polyclonal rabbit anti-mouse cleaved caspase-3 antibody overnight at 4°C, then stained with Alexa Fluor 488–conjugated anti-rabbit IgG and Alexa Fluor 594–conjugated anti-rat IgG (Molecular probes) at room temperature for 2 hours. For smooth muscle cells, carotid sections were incubated with polyclonal rabbit anti-mouse cleaved caspase-3 antibody overnight at 4°C, then stained with FITC-conjugated monoclonal anti-α-smooth muscle actin (Sigma-Aldrich) and Alexa Fluor 594–conjugated anti-rabbit IgG (Molecular probes) at room temperature for 2 hours. Finally, sections were stained with DAPI and fluorescence images acquired by confocal microscopy.

### Statistical Analysis

All data are expressed as mean±SEM (standard error of the mean). Comparisons between ligated (left) and nonligated (right) carotids for in situ and ex vivo fluorescence signal intensities were analyzed by the Wilcoxon signed-rank test. Comparisons of biodistribution of liver, spleen, kidney, and lung were analyzed by the unpaired Student *t* test, as they were normally distributed. Comparisons of cell number after in vitro photothermal therapy, in situ and ex vivo fluorescence signal intensities of left carotid arteries between ligation and sham group, intrinsic NIR signal intensities of ex vivo carotid arteries with and without SWNTs, and gallbladder biodistribution were analyzed by the Mann–Whitney *U* test. Comparisons among in vitro and ex vivo photothermal therapy groups were analyzed by the Kruskal–Wallis test with the Mann–Whitney *U* test with Bonferroni adjustment as the post hoc test. Comparisons of the area percentage of neointimal macrophage staining among photothermal therapy groups were analyzed by 2-way factorial analysis of variance (ANOVA). Comparison of serial FMT signal intensities was analyzed by 2-way repeated-measures ANOVA. *P*<0.05 was considered statistically significant.

## Results

### In Vitro Macrophage Uptake and Ablation

**Figure 1.**
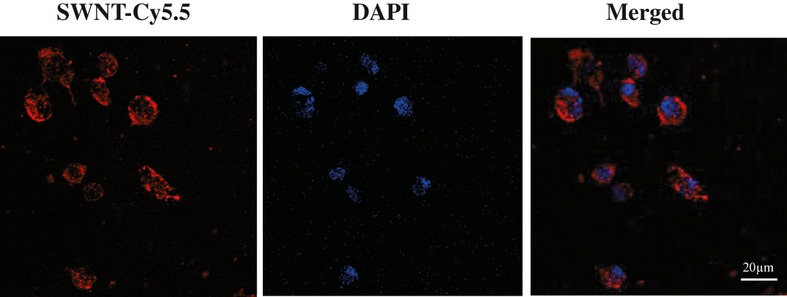
Confocal images of macrophages incubated with SWNTs. Cy5.5-conjugated SWNTs (red) can be seen in macrophages also stained with DAPI (blue) 24 hours after incubation. SWNT indicates single-walled carbon nanotubes.

**Figure 2.**
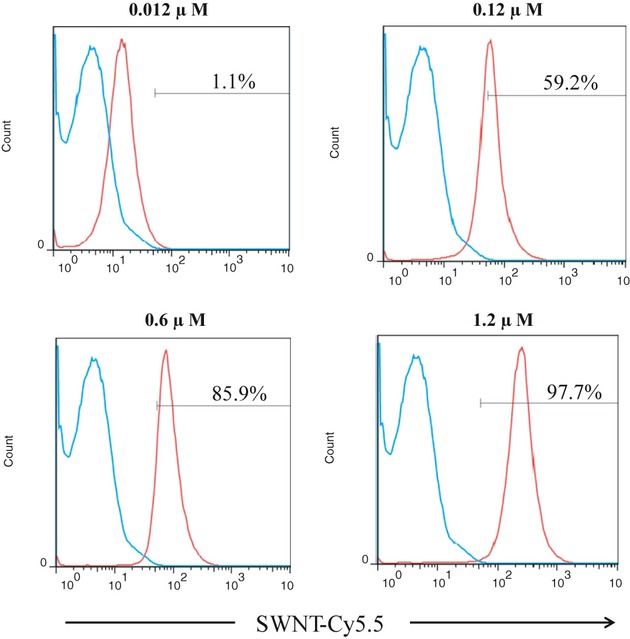
Flow cytometry showed SWNT uptake by macrophages in a concentration-dependent manner (0 to 1.2 μmol/L of Cy5.5). Blue lines indicate cells incubated with nonlabeled SWNTs. Red lines indicate cells incubated with Cy5.5-labeled SWNTs. SWNT indicates single-walled carbon nanotubes.

**Figure 3.**
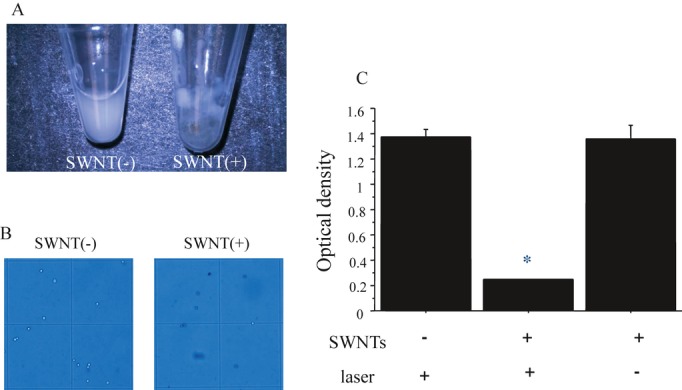
In vitro laser ablation. A, Photo of macrophage cell pellets with and without SWNTs after laser light exposure. Macrophages with SWNTs (right) were visibly disrupted during light exposure, but cells without SWNTs (left) showed no change. B, Representative images of trypan blue staining immediately after light exposure. The large majority of cells with SWNTs were dead after thermal ablation. C, Cell viability by MTT assay 24 hours after thermal ablation was significantly decreased in macrophages with SWNTs. *P<0.05 vs SWNT (−)/laser (+) and SWNT (+)/laser (−). SWNT indicates single-walled carbon nanotubes.

In vitro SWNT uptake by macrophages was clearly seen by confocal microscopy (Figure 1). The percent uptake of SWNTs by macrophages was 94±6%. Flow cytometry demonstrated that SWNTs were taken up by macrophages in a concentration-dependent manner (Figure 2). When exposed to 808-nm laser light, macrophages with SWNTs showed visible disruption (Figure 3A), whereas SWNT-free cells showed no significant change. Trypan blue staining after photothermal therapy showed a 93±3% reduction in viable macrophages incubated with SWNTs (0.07±0.05×10^7^ cells for SWNT[+] versus 1.0±0.03×10^7^ cells for SWNT[−], *P*<0.03; Figure 3B). Further quantifying cell viability at 24 hours showed a similar effect of SWNTs (0.2±0.002 absorbance units [AU] for SWNT[+]/light[+] versus 1.4±0.03 AU for SWNT[−]/light[+] and 1.4±0.06 AU for SWNT[+]/light[−], *P*<0.05; Figure 3C).

### Fluorescence Imaging of SWNTs

**Figure 4.**
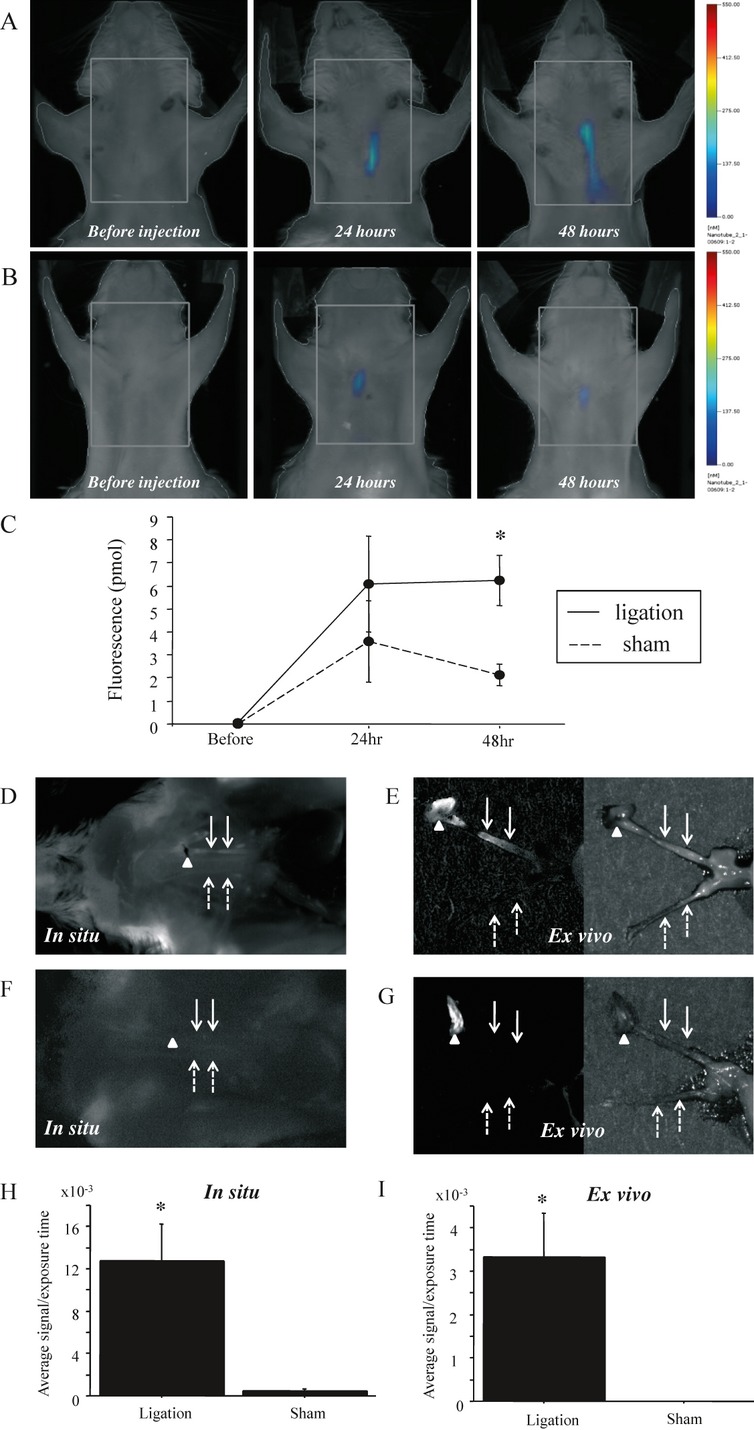
Fluorescence imaging of mouse carotid arteries after administration of SWNTs. A and B, Representative serial in vivo FMT images of a carotid-ligated mouse (A) and a sham-operated mouse (B) before and after injection of Cy5.5-conjugated SWNTs. The limited, transient signal in sham mice may represent some inflammation near the carotid from the surgical procedure. C, Quantitative analysis of signal intensities showed significantly higher signal in the ligation group over time; *P<0.05 vs sham. D and E, Representative in situ (D) and ex vivo (E) fluorescence images of a carotid-ligated mouse 48 hours after injection of Cy5.5-conjugated SWNTs. Both in situ and ex vivo images exhibited SWNT accumulation in ligated left carotid (solid arrows), but not contralateral nonligated right carotid (dashed arrows). Arrowhead shows the suture. F and G, Representative in situ (F) and ex vivo (G) fluorescence images of a sham-operated mouse 48 hours after injection of Cy5.5-conjugated SWNTs. Both in situ and ex vivo images exhibited minimal SWNT accumulation in the left (solid arrows) and right (dashed arrows) carotid arteries. Arrowhead shows the signal from the suture in both the ligated and sham images. Note that exposure time was longer in the sham images because of lower Cy5.5 signal, resulting in a darker background. H and I, Quantitative analysis of signal intensities of left carotid arteries of ligation and sham groups. The SWNT signal intensities of carotid-ligated mice in both in situ and ex vivo images were significantly greater than in sham-operated mice; *P<0.01 vs sham. SWNT indicates single-walled carbon nanotubes; FMT, fluorescence molecular tomography.

In vivo FMT images exhibited signal enhancement of the left carotid artery 24 and 48 hours after injection of Cy5.5-conjugated SWNTs in ligated mice (Figure 4A), with a limited transient signal seen in sham-operated mice (Figure 4B). Signal intensity over time was significantly higher in ligation compared with sham (Figure 3C, *P*<0.05). As shown in Figure 4D and 4E, both in situ and ex vivo images at 48 hours exhibited an enhanced fluorescence signal localized to the ligated left carotid arteries. There was minimal evidence of SWNT accumulation in sham-operated mice (Figure 4F and 4G) or in contralateral nonligated right carotid arteries (dashed arrows in Figure 4D through 4G). Quantitative analysis of the signal intensity (Figure 4H and 4I) showed that the ligated left carotid arteries had significantly higher signal than did sham-operated left carotid arteries (in situ: 13±3.5×10^−3^ versus 0.5±0.2×10^−3^ counts/second, *P*<0.01; ex vivo: 3.3±1.0×10^−3^ versus 0.1±0.1×10^−6^ counts/second, *P*<0.01).

**Figure 5.**
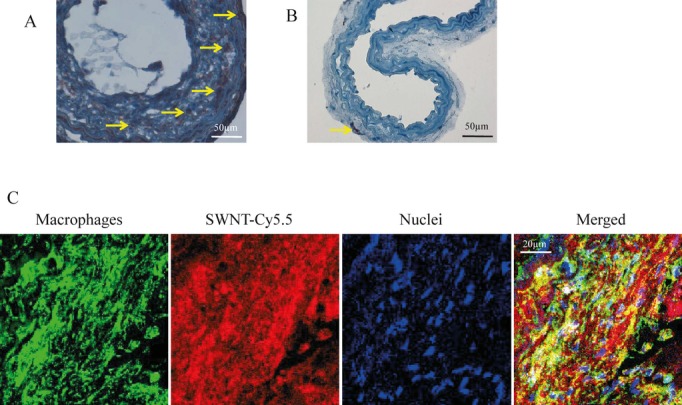
Immunostaining of carotid arteries after administration of Cy5.5-conjugated SWNTs. A and B, Immunohistochemistry for macrophages in ligated left carotid artery (A) and sham-operated left carotid artery (B). Ligated left carotid artery showed numerous macrophages infiltrating the neointima and adventitia (red staining shown by arrows). Sham-operated left carotid artery did not show neointimal formation, and very few macrophages were seen in the adventitia. Original magnification ×400. C, Immunofluorescence staining exhibited good but not complete colocalization (yellow) of Cy5.5-conjugated SWNTs (red) with macrophages (green) in the neointima. Original magnification ×630. SWNT indicates single-walled carbon nanotubes.

Immunohistochemistry for macrophages demonstrated substantial infiltration of macrophages in the neointima and adventitia in ligated left carotid arteries (Figure 5A). In contrast, only a small number of macrophages were seen in the adventitia of sham-operated left carotid arteries (Figure 5B). Immunofluorescence staining exhibited colocalization of SWNT-Cy5.5 and macrophages in the neointima (Figure 5C).

Table 1 shows the biodistribution of SWNT-Cy5.5 in various organs of ligation and sham groups at 48 hours. The liver exhibited higher signal intensity than did other organs. The signal intensity of each organ (liver, spleen, lung, gallbladder, and kidney) did not differ significantly between the 2 groups.

**Table 1.** Biodistribution of SWNTs (average signal/exposure time in counts/second)

	Ligation (n=4)	Sham (n=4)	*P*
Liver	0.125±0.019	0.096±0.012	0.24

Spleen	0.017±0.006	0.005±0.001	0.12

Gallbladder	0.014±0.001	0.011±0.004	0.29

Kidney	0.014±0.001	0.014±0.003	0.99

Lung	0.002±0.0001	0.002±0.0002	0.71

SWNT indicates single-walled carbon nanotubes.

### Intrinsic Near-Infrared Imaging of SWNTs

**Figure 6.**
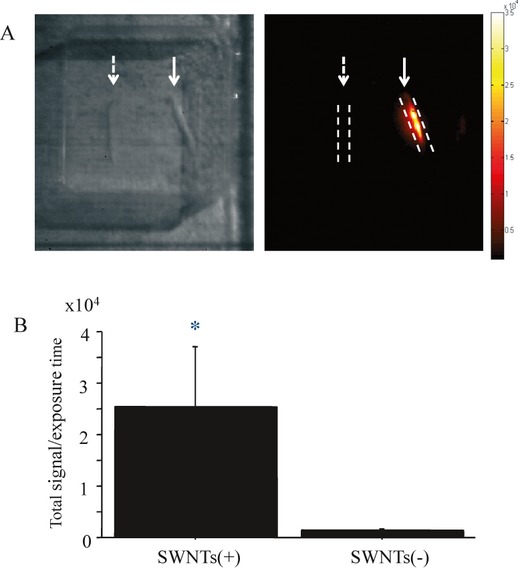
Intrinsic SWNT fluorescence imaging of carotids. A, White light image (left) and NIR image (right) of ex vivo ligated left carotid arteries. The ligated left carotid artery from a mouse injected with SWNTs (solid arrows) showed high NIR signal because of SWNT accumulation, but the ligated left carotid artery from a mouse without SWNTs injected did not show similar NIR signal (dashed arrows). B, Quantitative analysis of NIR signal intensity showed significantly higher signal in ligated left carotid arteries with SWNTs; *P<0.01 vs SWNTs(−). SWNT indicates single-walled carbon nanotubes; NIR, near-infrared.

NIR imaging of intrinsic SWNT fluorescence showed high signal in ex vivo ligated left carotid arteries from mice receiving SWNTs but no significant signal in ligated left carotid arteries without SWNTs (Figure 6A). Quantitative analysis demonstrated the significantly greater NIR signal intensity in ligated left carotid arteries with SWNTs (2.5±1.2×10^4^ versus 0.1±0.03×10^4^ counts/ms, *P*<0.01; Figure 6B).

### Photothermal Ablation of Vascular Macrophages

**Figure 7.**
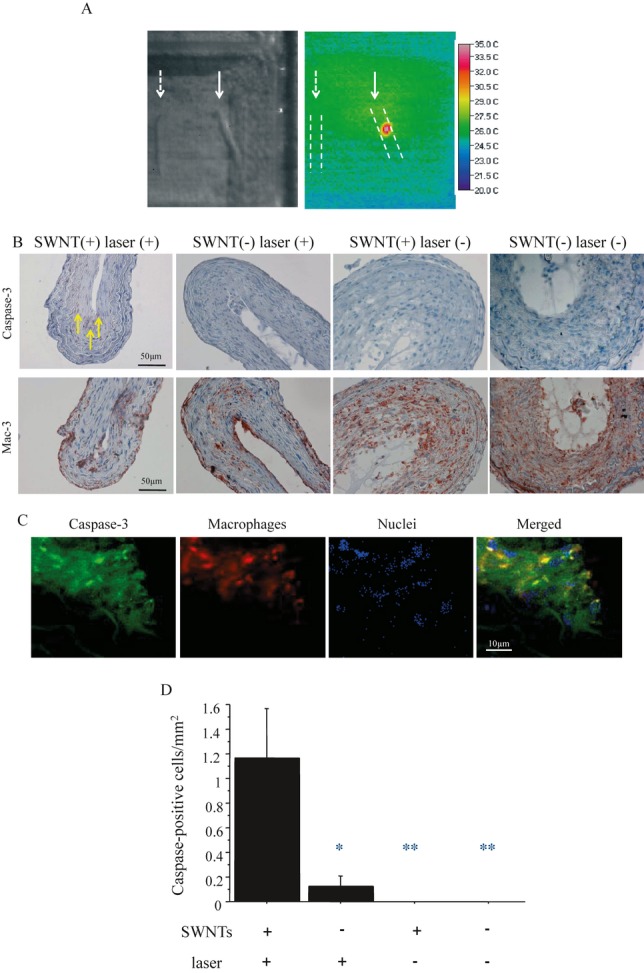
Ex vivo thermal ablation. A, White light image (left) and thermal image (right) of the same specimen as Figure 6A demonstrated temperature increase of the ligated left carotid artery with SWNTs (solid arrow), but not the ligated left carotid artery without SWNTs (dashed arrow). Dashed lines show outline of carotid arteries. B, Immunohistochemistry for caspase-3 (upper) and macrophages (lower). Thermal ablation induced apoptosis in ligated left carotid arteries with SWNTs, with caspase-3-positive cells corresponding to macrophages (arrows). Minimal apoptosis induction was noted in light-exposed ligated left carotid arteries without SWNTs or in ligated left carotid arteries not exposed to laser light, with or without SWNTs. Original magnification ×400. C, Immnunnofluorescence staining for caspase-3 and macrophages confirmed the colocalization (yellow) of macrophages (red) and caspase-3-positive cells (green). Original magnification ×1000. D, Quantitative analysis of the number of caspase-3-positive cells showed a significant increase only in ligated carotids with SWNTs plus laser light exposure; *P<0.02 vs SWNTs (+)/laser (+), **P<0.007 vs SWNTs (+)/laser (+). SWNT indicates single-walled carbon nanotubes.

Simultaneous laser light exposure of freshly excised left carotid arteries with and without SWNTs showed a temperature increase only of carotids with SWNTs (Figure 7A). Immunohistochemistry showed that photothermal therapy induced apoptosis in the neointima and adventitia of ligated left carotid arteries given SWNTs (Figure 7B), with caspase-3-positive cells corresponding to macrophages (Figure 7B). Minimal apoptosis induction was noted after laser light exposure of ligated left carotid arteries without SWNTs or in ligated left carotid arteries not exposed to laser light, with or without SWNTs (Figure 7B).

**Figure 8.**
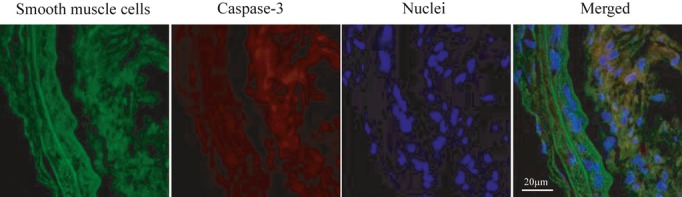
Immunofluorescence staining exhibited minimal colocalization (yellow) of caspase-3 positive cells (red) with smooth muscle cells (green) in the neointima. Original magnification ×630.

**Figure 9.**
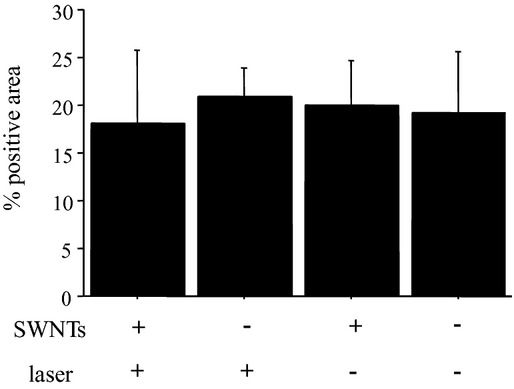
Quantitative analysis of the area percentage of neointimal macrophage staining showed no significant differences between groups (*P*=0.5). SWNT indicates single-walled carbon nanotubes.

Confocal microscopy further confirmed the colocalization of macrophages and caspase-3-positive cells (Figure 7C), with minimal colocalization of smooth muscle cells and caspase-3-positive cells (Figure 8). Quantitative analysis of the number of caspase-3-positive cells showed significantly greater apoptosis in light-exposed left carotid arteries with SWNTs compared with all 3 control groups (Figure 7D). All 4 groups had a similar proportion of macrophages by quantitative analysis (*P*=0.5, Figure 9).

## Discussion

SWNTs were taken up by macrophages both in cell culture and in ligated murine carotid arteries, allowing both Cy5.5-based fluorescence imaging and intrinsic NIR imaging. Furthermore, combining SWNTs with laser light exposure caused thermal ablation of macrophages in vitro and in freshly excised ligated carotids. To the best of our knowledge, this study is the first to show that carbon nanotubes allow imaging and thermal ablation of vascular macrophages.

SWNTs have a number of valuable properties for molecular and cellular imaging and therapy. Their nanosize has made them amenable to cellular binding and/or uptake, and their functionality can be enhanced with the attachment of NIR fluorophores and/or targeting ligands (eg, RGD, folate).^6,22–24^ We found high uptake of nontargeted SWNTs in vascular macrophages in this study, but further enhancement of macrophage uptake with specific targeting ligands may be beneficial.^19,25,26^

SWNTs can be used for molecular and cellular imaging by attaching a fluorophore^6,27^ or through their recently described intrinsic photoluminescent properties.^16,28–30^ In this study, we showed that either approach can be used. The high performance of the intrinsic NIR imaging capability of SWNTs shown in vivo in cancer^10^ holds promise for in vivo vascular imaging. More data are needed on in vivo sensitivity of intrinsic SWNT fluorescence for imaging vascular inflammation, as this can be challenging in deeper vessels and larger species. FMT is a useful modality for noninvasive whole-body imaging in mice, but anatomic registration is challenging. Hybrid imaging protocols, such as FMT-CT, may improve anatomic localization of signals in vivo.^21,31^

Heating of SWNTs in response to laser light excitation has shown promise for photothermal ablation of cancer cells,^6,8,10^ with strong optical absorbance demonstrated in the NIR range^6^ and specific testing for heating and nontoxicity at 808 nm.^6^ NIR light is known to penetrate up to 10 cm through certain tissues, even using low (FDA Class 1) microwatt lasers.^32^ SWNTs are potentially advantageous for photothermal ablation, as they have shown tumor elimination with 10-fold-lower injected doses and lower laser power compared with gold nanorods.^10^ In this study, side-by-side external laser light exposure of diseased carotid specimens with and without SWNTs showed heating and macrophage apoptosis only in the presence of SWNTs. SWNTs alone did not induce apoptosis, nor did laser exposure alone. We have not observed significant acute or chronic toxicity of these SWNTs with regard to clinical and laboratory parameters, histology, or survival in mice followed 3 to 5 months after injection,^10,33^ but longer-term studies are needed. Although quantitative analysis of carotid temperature change was not performed, the goal was not to ablate the entire vessel, only the SWNT-containing macrophages. The colocalization of apoptosis and macrophage staining was supportive of achieving this goal, particularly as monitoring the temperature of individual macrophages within the specimen was not feasible.

This macrophage photothermal ablation approach to treat atherosclerosis has both promise and challenges. One advantage is that it requires a double hit—cellular uptake of SWNTs and local laser light excitation—which provides a high degree of specificity. Macrophage-specific uptake and ablation could be further enhanced with targeted SWNTs.^19,20,23–26^ Alternative therapeutic approaches, such as toxin-loaded targeted nanoparticles, may be prone to uptake in other organs with resulting collateral damage (particularly liver and other components of the reticuloendothelial system). Similarly, direct thermal ablation (eg, focused ultrasound) is difficult to target to specific cells. Of course, clinical translation of this approach in vivo has multiple challenges, including the temperature modulating effect of circulating blood and the need for an intravascular laser light delivery system for deeper vessels, which has been used previously.^11,12,15^ Furthermore, although reduction of plaque macrophage burden and plaque area has been shown with phototoxic agents,^11^ thermal macrophage ablation with SWNTs could potentially lead to an increase in vascular inflammation. There may also be heterogeneity of the photothermal response, because of the complexity of human plaque tissue, variable macrophage uptake of SWNTs, and the heat-dissipating effect of flowing blood near the lumen. In vivo animal testing and serial monitoring of disease response will be needed to determine the potential for clinical translation.

In conclusion, we have demonstrated that carbon nanotubes are capable of both fluorescence imaging and photothermal ablation of vascular macrophages. Further development and testing of an in vivo vascular photothermal ablation system are warranted. Thus, carbon nanotubes are promising “theranostic” agents for vascular inflammation and atherosclerosis.
